# Synthesis, crystal structure and thermal properties of a new polymorphic modification of diiso­thio­cyanato­tetra­kis­(4-methyl­pyridine)cobalt(II)

**DOI:** 10.1107/S2056989024004997

**Published:** 2024-05-31

**Authors:** Christian Näther, Aleksej Jochim

**Affiliations:** a Institut für Anorganische Chemie, Universität Kiel, Germany; Tokyo University of Science, Japan

**Keywords:** synthesis, discrete complex, polymorphism, thermal properties, cobalt thio­cyanate, 4-methyl­pyridine, crystal structure

## Abstract

The title compound, [Co(NCS)_2_(C_6_H_7_N)_4_] or Co(NCS)_2_(4-methyl­pyridine)_4_, was prepared by the reaction of Co(NCS)_2_ with 4-methyl­pyridine in water and is isotypic to one of the polymorphs of Ni(NCS)_2_(4-methyl­pyridine)_4_ [Kerr & Williams (1977 ▸). *Acta Cryst.* B**33**, 3589–3592 and Soldatov *et al.* (2004 ▸). *Cryst. Growth Des.* **4**, 1185–1194]. Comparison of the experimental X-ray powder pattern with that calculated from the single-crystal data proves that a pure phase has been obtained. The asymmetric unit consists of one Co^II^ cation, two crystallographically independent thio­cyanate anions and four independent 4-meth­yl­pyridine ligands, all located in general positions. The Co^II^ cations are sixfold coordinated to two terminally N-bonded thio­cyanate anions and four 4-methyl­pyridine coligands within slightly distorted octa­hedra. Between the complexes, a number of weak C—H⋯N and C—H⋯S contacts are found. This structure represent a polymorphic modification of Co(NCS)_2_(4-methyl­pyridine)_4_ already reported in the CCD [Harris *et al.* (2003 ▸). *NASA Technical Reports*, 211890]. In contrast to this form, the crystal structure of the new polymorph shows a denser packing, indicating that it is thermodynamically stable at least at low temperatures. Thermogravimetric and differential thermoanalysis reveal that the title compound starts to decomposes at about 100°C and that the coligands are removed in separate steps without any sign of a polymorphic transition before decomposition.

## Abstract 2

The crystal structure of title compound consists of discrete complexes in which the Co^II^ cations are octa­hedrally coordinated to two terminally N-bonded thio­cyanate anions and four 4-methyl­pyridine coligands and represents a new polymorphic modification of Co(NCS)_2_(4-methyl­pyridine)_4_, which is already reported in the literature.

## 1. Chemical context

Polymorphism is a widespread phenomenon and of equal importance in academic and industrial research. It is frequently found in organic compounds but there are also several examples where it is observed in coordination compounds (Moulton & Zaworotko, 2001 ▸; Braga & Grepioni, 2000 ▸; Tao *et al.*, 2012 ▸). This is the case, for example, for coordination compounds based on thio­cyanate anions, which we have been inter­ested in for several years. The majority of polymorphic modifications in this class of compounds are observed for discrete complexes with terminally N-bonded ligands (Wöhlert *et al.*, 2013 ▸; Neumann *et al.*, 2018*a* ▸). In contrast, compounds with a bridging coordination of the anionic ligands typically form isomeric modifications (Mautner *et al.*, 2018 ▸; Neumann *et al.*, 2018*b* ▸; Böhme *et al.*, 2020 ▸; Jochim *et al.*, 2018 ▸). Within this project, we are especially inter­ested in compounds based on Co(NCS)_2_ which, because if its high magneticotropy, shows a versatile magnetic behavior (Rams *et al.*, 2017 ▸, 2020 ▸). In the course of these investigations, we became inter­ested in 4-methyl­pyridine as coligand, with a special focus on Co(NCS)_2_ compounds.

Several compounds based on Co(NCS)_2_ have already been reported with this ligand, predominantly discrete complexes with a tetra­hedral or an octa­hedral coordination, with most of them forming clathrates (see *Database survey*). As part of our synthetic work we have obtained crystals that were characterized by single-crystal X-ray diffraction. This proves that a discrete complex with the composition Co(NCS)_2_(4-methyl­pyridine)_4_ was obtained. Based on these findings, a CSD search was performed, which revealed that the structure of a compound with this composition had already been reported by Solacolu and co-workers and Harris and co-workers [refcodes QQQGKG (Solacolu *et al.*, 1974 ▸) and VERNUC (Harris *et al.*, 2003 ▸)]. The title compound crystallizes differently, which means that we have obtained a new polymorphic modification of this complex. 
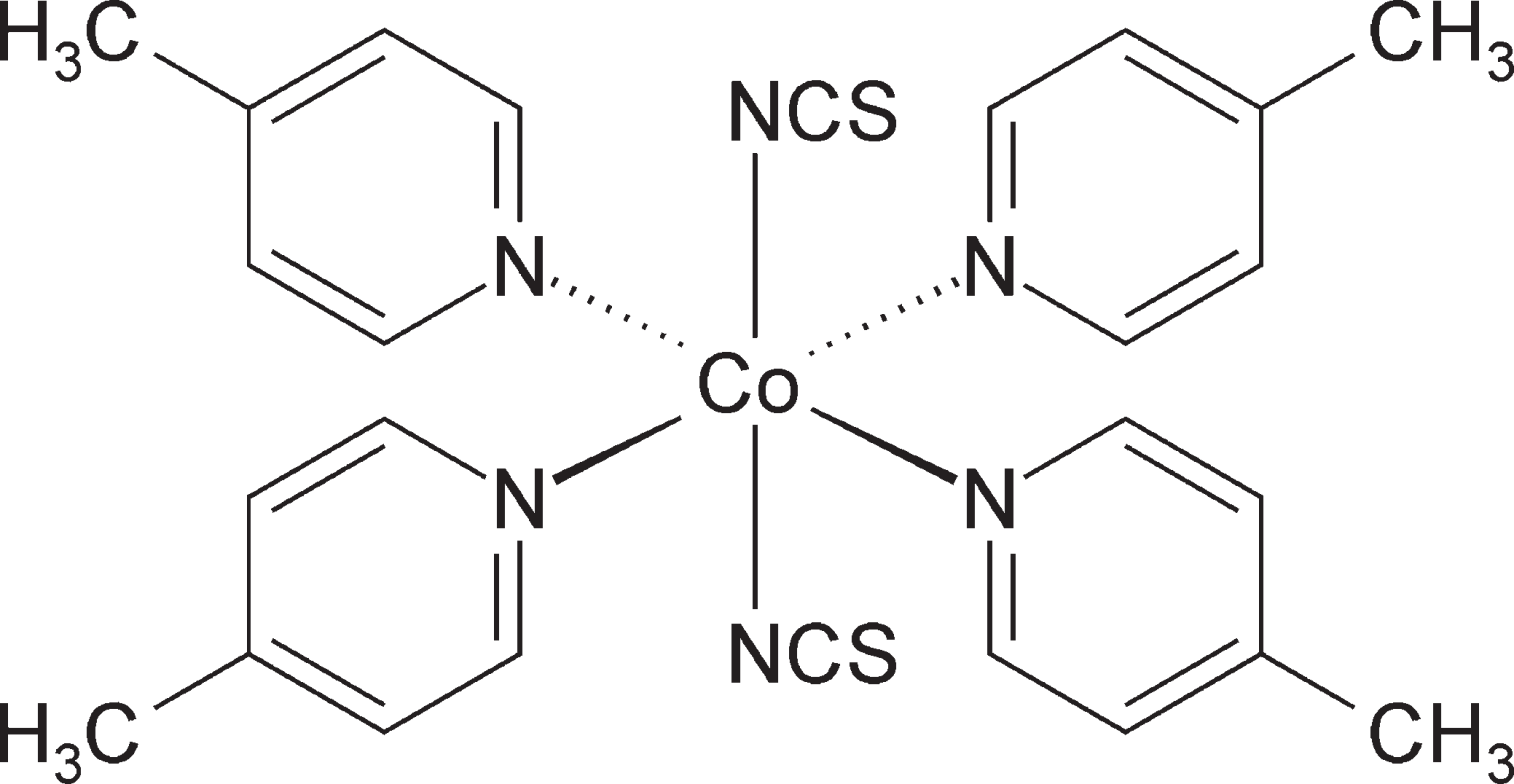


## 2. Structural commentary

**Figure 1:**
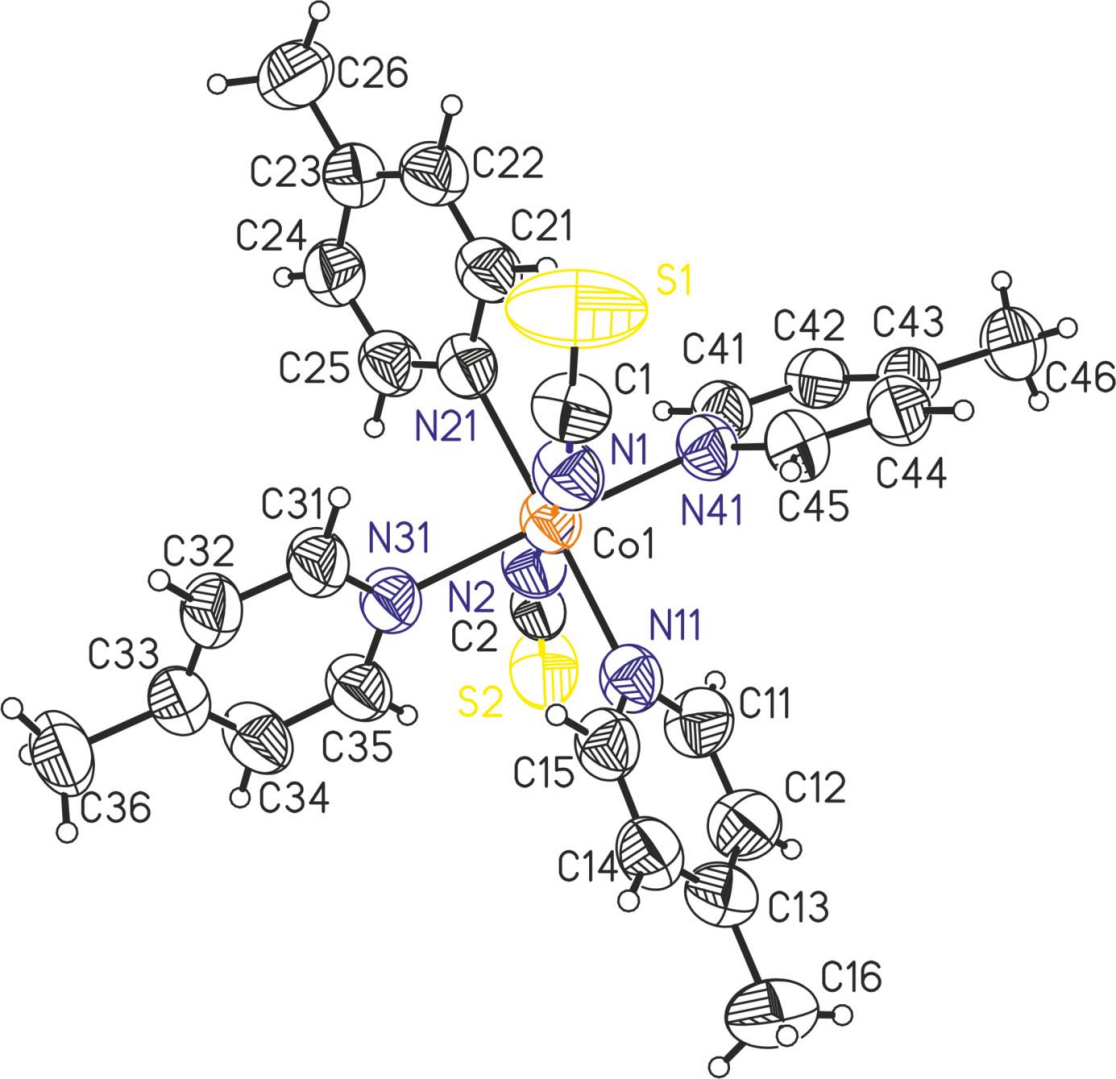
Crystal structure of the title compound with atom labeling and displacement ellipsoids drawn at the 50% probability level.

The title compound, Co(NCS)_2_(4-methyl­pyridine)_4_, is isotypic to Ni(NCS)_2_(4-methyl­pyridine)_4_ already reported in the literature (refcode ICMPNI01; Kerr & Williams, 1977 ▸ and Soldatov *et al.*, 2004 ▸). Its asymmetric unit consists of one Co^II^ cation, two thio­cyanate anions and four 4-methyl­pyridine coligands that are located in general positions (Fig. 1 ▸). The metal cations are sixfold coordinated to two terminally N-bonded thio­cyanate anions and four 4-methyl­pyridine co­ligands into discrete complexes. Bond lengths and angles are comparable to those in the polymorphic modification already reported in the literature (refcode VERNUC; Harris *et al.*, 2003 ▸) and show that a slightly distorted octa­hedral coordination is present (Table 1 ▸).

The title compound represents a further polymorph of the modifications that have already been reported in the literature [refcodes QQQGKG (Solacolu *et al.*, 1974 ▸) and VERNUC (Harris *et al.*, 2003 ▸)], but it is noted that some contradictory results have been published. The modification reported by Harris and co-workers crystallizes in the tetra­gonal space group *I*4_1_/*a* with eight formula units in the unit cell and a unit-cell volume of 6329.415 Å^3^. The form reported by Solacolu and co-workers crystallizes in the space group *I*4_1_/*a* but with twelve formula units in the unit cell with a unit-cell volume of 6877.013 Å^3^. However, in the same paper they also present a *p*-xylene clathrate crystallizing in the same space space group with a unit-cell volume of 6324.998 Å^3^, which is very similar to that in the modification reported by Harris *et al.* It is therefore likely that the two unit-cell volumes were accidentally mixed up and that only one modification of Co(NCS)_2_(4-methyl­pyridine)_4_ is reported. This is further supported by the fact that in the form reported by Solacula *et al.* with *Z* = 12, each non-hydrogen atom would need a volume of 16.4 Å^3^, which seem to be much too low for such a complex. Unfortunately, no atomic coordinates are given for the ansolvate and the solvate reported by Solacula *et al.* and therefore those crystal structures cannot be compared with that of the form reported by Harris *et al.*

However, if the volume of each non hydrogen atom in the title compound (20.3 Å^3^) is compared with that of the modification reported by Harris *et al.* (22.6 Å^3^), it is obvious that the title compound is much more densely packed, indicating that this modification represents the thermodynamically stable form, at least at 0 K.

## 3. Supra­molecular features

**Figure 2:**
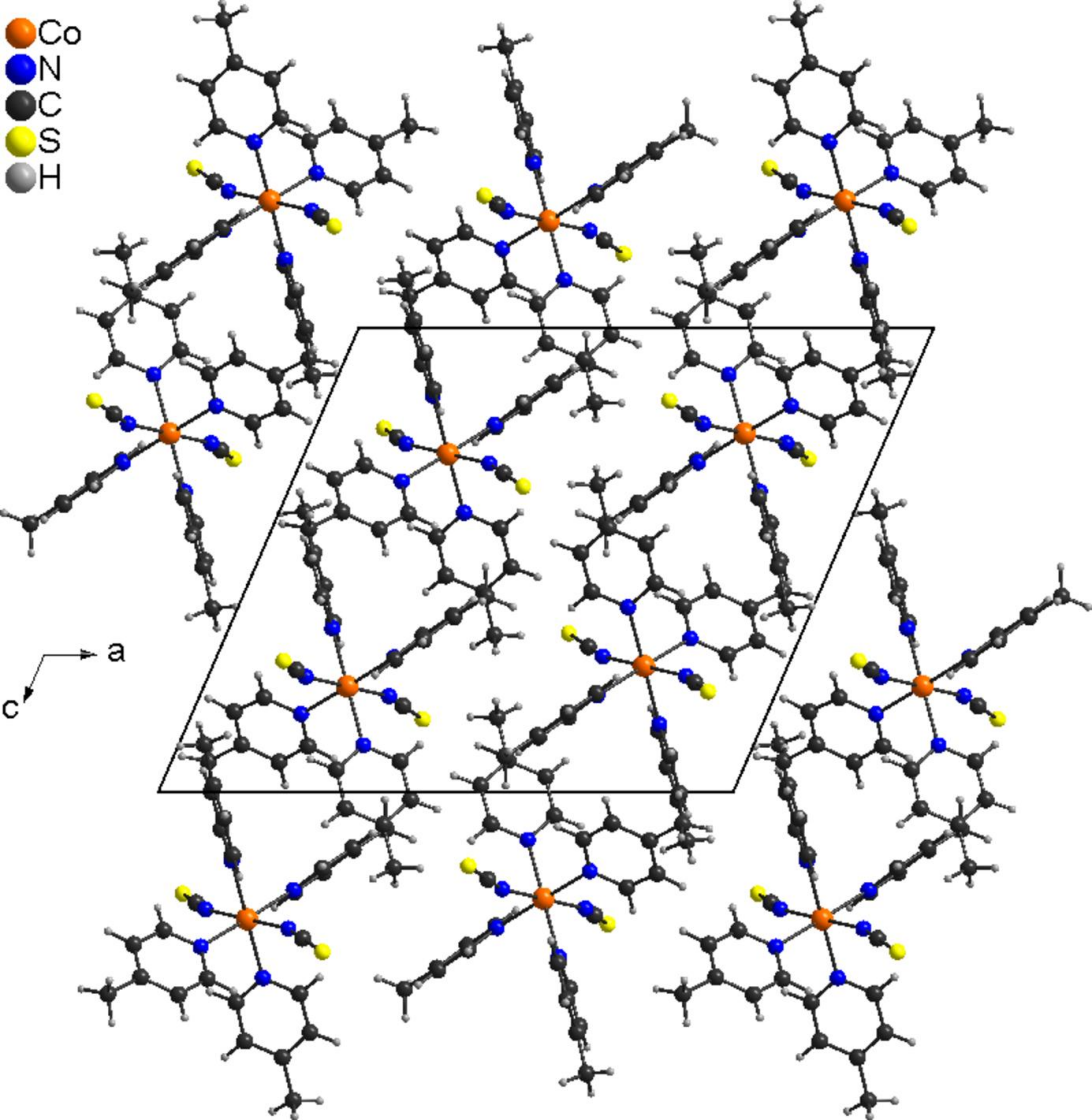
Crystal structure of the title compound in a view along the crystallographic *b-*axis direction.

In the crystal structure of the title compound, the discrete complexes are arranged in columns that propagate along the crystallographic *b*-axis direction (Fig. 2 ▸). A number of C—H⋯N and C—H⋯S contacts are observed between the complexes, but from the H⋯N and H⋯S distances and the C—H⋯N and C—H⋯S angles (Table 2 ▸) it is unlikely that these are significant inter­actions.

**Figure 3:**
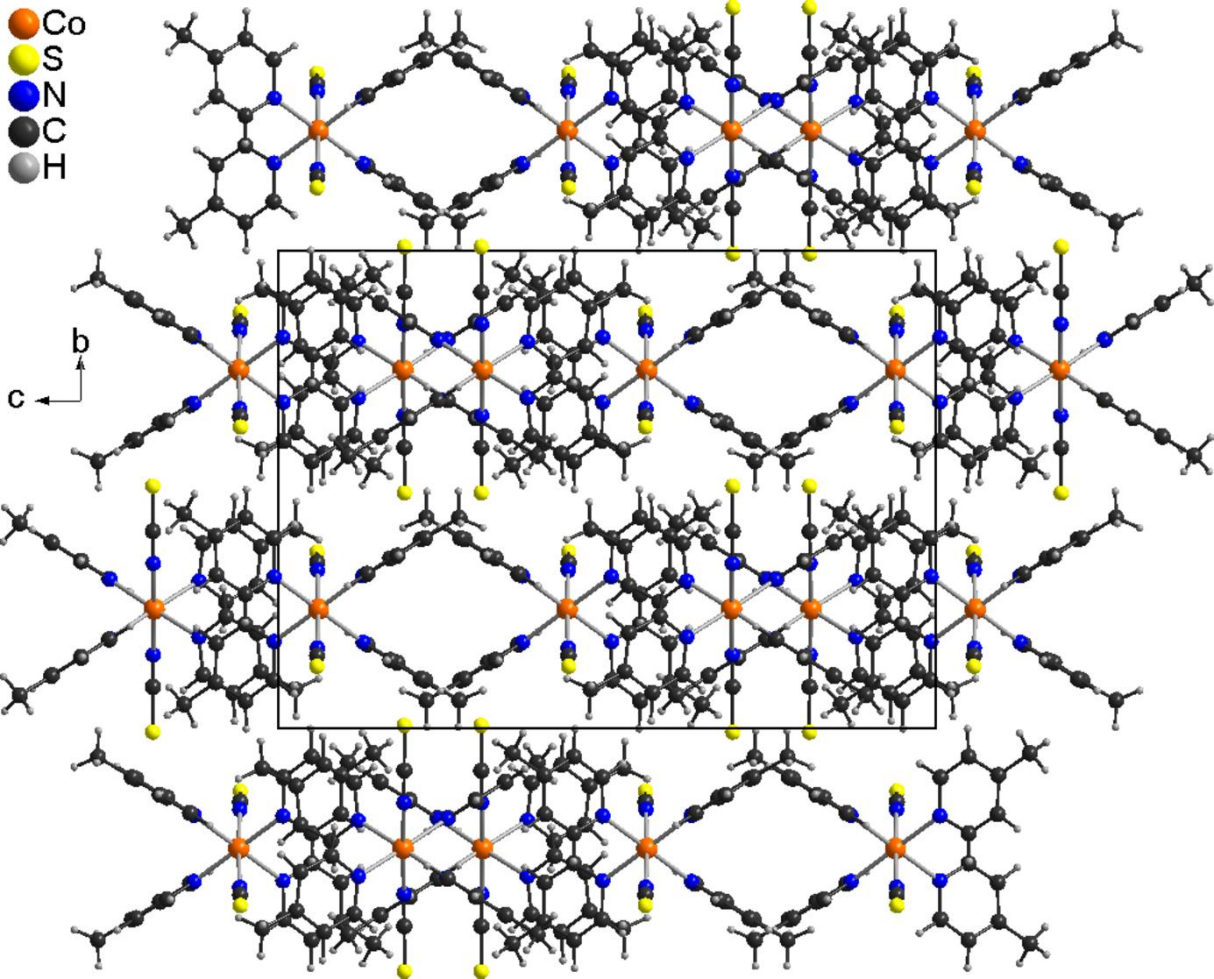
Crystal structure of Co(NCS)_2_(4-methyl­pyridine)_4_ (reported by Harris *et al.*, 2003 ▸) drawn from the CIF file available in the CSD. Note that this structure contains three-dimensional pores in which solvents might be incorporated.

In contrast, the form reported by Harris *et al.*, exhibits three-dimensional pores (Fig. 3 ▸), which might be responsible for the low density of this modification. Moreover, because most clathrates are isotypic to the form reported by Harris *et al.*, it is possible that these solvates lose their solvent mol­ecules and transform into the ansolvate, presumably without collapse of the overall structure.

## 4. Database survey

A search of the CSD (version 5.43, last update December 2024; Groom *et al.*, 2016 ▸) using CONQUEST (Bruno *et al.*, 2002 ▸) reveals that ten compounds with Co(NCS)_2_ and 4-methyl­pyridine are present in the CSD. This includes two discrete complexes with a tetra­hedral coordination and the composition Co(NCS)_2_(4-methyl­pyridine)_2_ and Co(NCS)_2_(4-methyl­pyridine)_2_ *p*-xylene clathrate (refcodes QQQGKD and QQQGKJ; Solacolu *et al.*, 1974 ▸). There is also one compound reported with the composition Co(NCS)_2_(4-methyl­pyridine)_2_·2*p*-toluidine clathrate in which the cations are linked into chains (refcode CECDAP; Micu-Semeniuc *et al.*, 1983 ▸).

All remaining compounds consists of discrete complexes with the composition Co(NCS)_2_(4-methyl­pyridine)_4_, [refcodes QQQGKG (Solacolu *et al.*, 1974 ▸) and VERNUC (Harris *et al.*, 2003 ▸)] with some of them crystallizing as clathrates [*p*-toluidine clathrate (CECCOC; Micu-Semeniuc *et al.*, 1983 ▸), *p*-xylene clathrate (QQQGKJ; Solacolu *et al.*, 1974 ▸), 4-methyl­pyridine clathrate (XIHHEB, Harris *et al.*, 2001 ▸, and XIHHEB01, Harris *et al.*, 2003 ▸), nitro­benzene, nitro­ethane and benzene clathrate (ZZZUXU, ZZZUXY and ZZZUYI; Belitskus *et al.*, 1963 ▸)].

Finally, it is noted that for Ni(NCS)_2_(4-methyl­pyridine)_4_, two different polymorphic modifications have also been reported, including two reports on the form that is isotypic to the title compound [refcodes ICMPNI01 (Kerr & Williams, 1977 ▸) and ICMPNI03 (Soldatov *et al.*, 2004 ▸)] and four reports on the form isotypic to Co(NCS)_2_(4-methyl­pyridine)_4_ [ICMPNI (Andreetti *et al.*, 1972 ▸), ICMPNI02 (Harris *et al.*, 2001 ▸) ICMPNI04 and ICMPNI05 (Soldatov *et al.*, 2004 ▸) and ICMPNI06 (Harris *et al.*, 2003 ▸)].

## 5. Additional investigations

**Figure 4:**
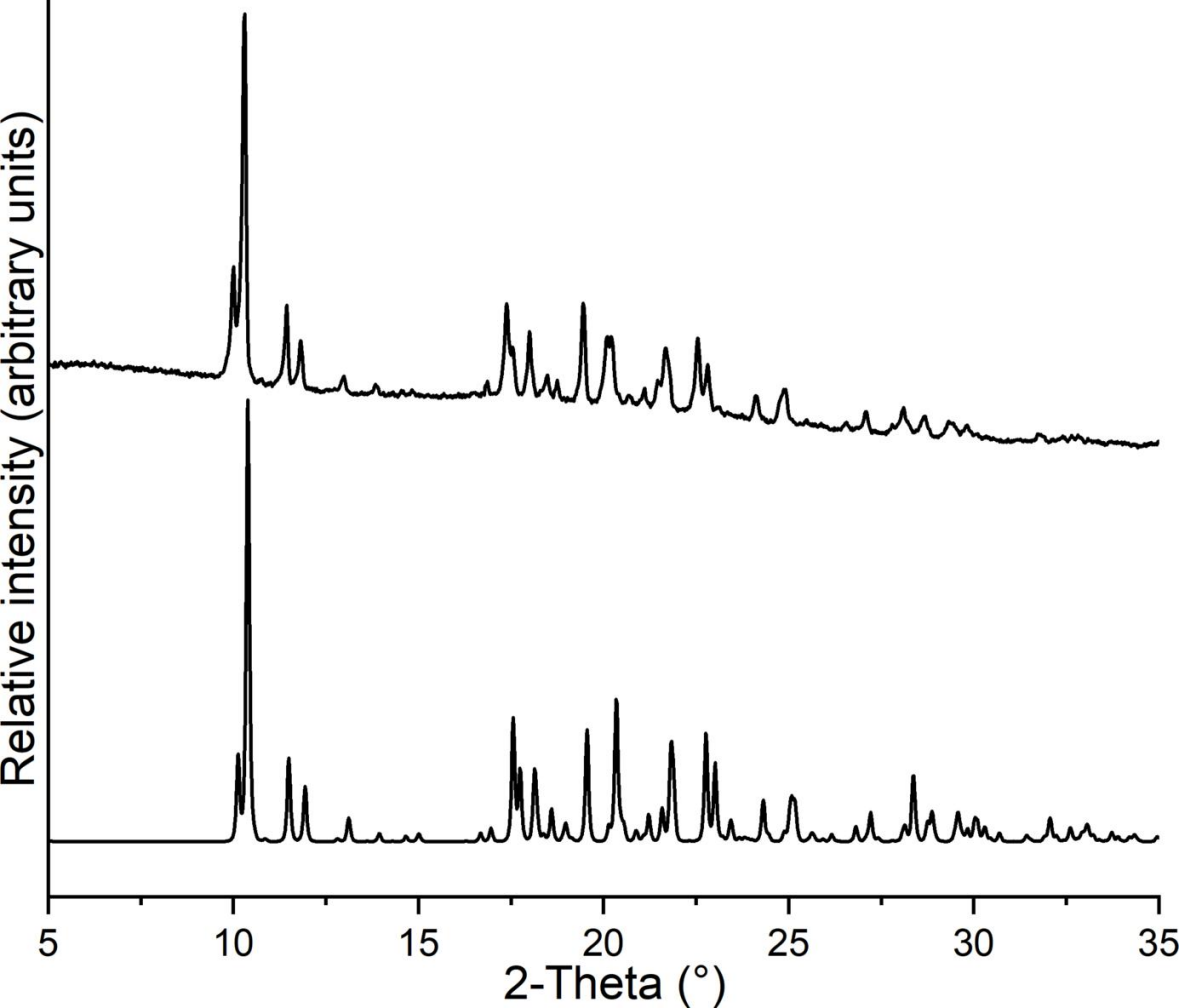
Experimental (top) and calculated (bottom) X-ray powder patterns of the title compound.

The experimental X-ray powder pattern of the title compound was compared with that calculated from single-crystal data; this proves that a pure crystalline phase has been obtained (Fig. 4 ▸).

**Figure 5:**
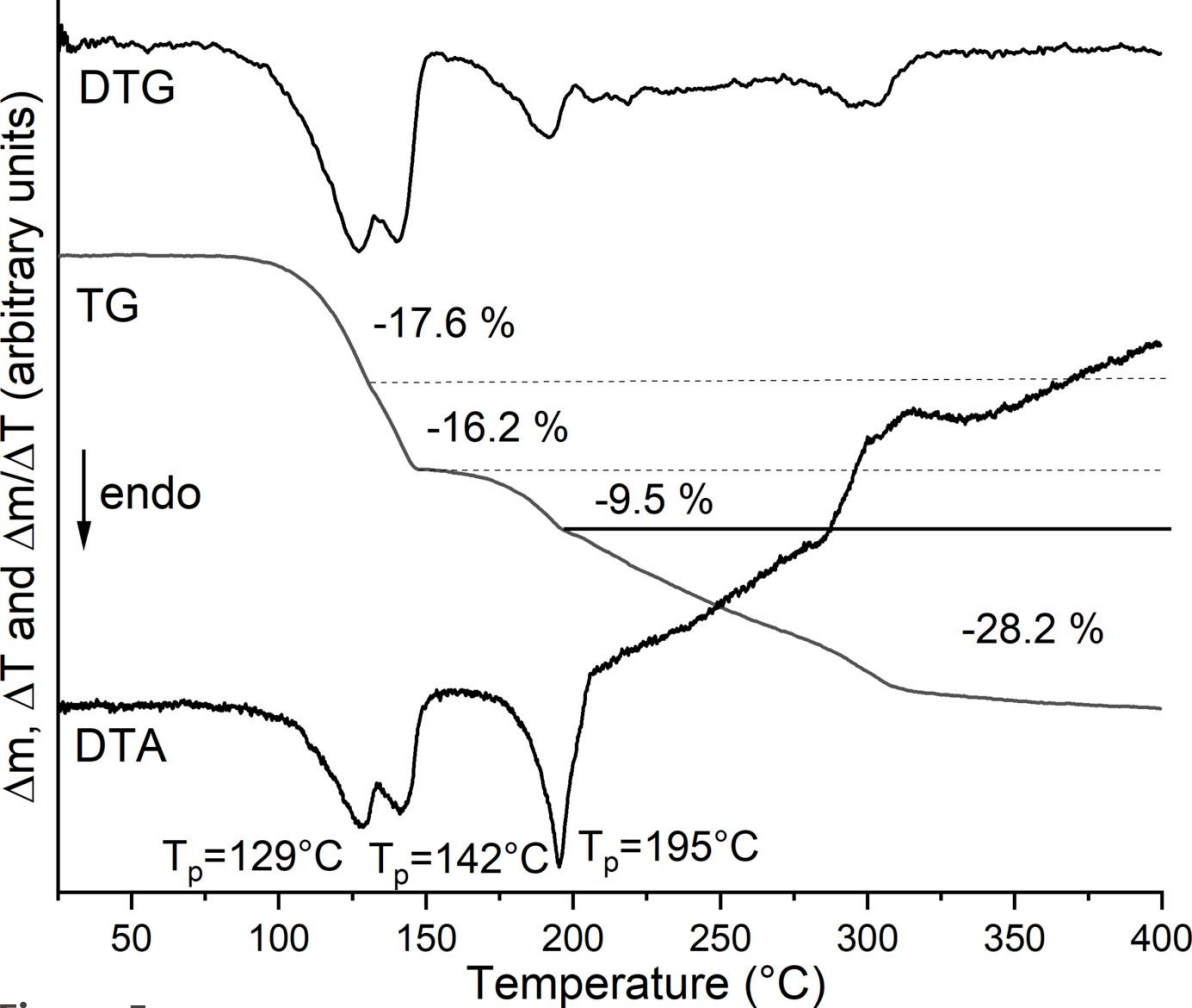
DTG, TG and DTG curves for the title compound. The mass loss is given in % and the peak temperature in °C.

The title compound was also investigated by thermogravimetry and differential thermoanalysis (TG-DTA) measurements. Upon heating, several mass losses are observed, which are accompanied by endothermic events in the DTA curve (Fig. 4 ▸). From the DTG curve, it is obvious that all mass losses are poorly resolved (Fig. 5 ▸). The experimental mass loss of the first and second step is in rough agreement with that calculated for the removal of one 4-methyl­pyridine ligand in each step (Δ*m*_calc._ = 17.0%). Upon further heating, the remaining 4-methyl­pyridine ligands are removed and the Co(NCS)_2_ formed as an inter­mediate decomposes.

## 6. Synthesis and crystallization

**Synthesis**

Co(NCS)_2_ (99.9%) and 4-methyl­pyridine (98%) were purchased from Sigma Aldrich. Single crystals of the title compound suitable for structure determination were obtained by dissolving 0.25 mmol (43.8 mg) of Co(NCS)_2_ in 7 mL of demineralized water. To this solution, 1.00 mmol (97.3 µl) of 4-methyl­pyridine were added and the reaction mixture was heated to 413 K for 15 min in a closed vial. Afterwards, it was cooled to 363 K and stored at this temperature overnight, leading to the formation of violet-colored crystals. Larger amounts of a crystalline powder were prepared by stirring 0.50 mmol (87.6 mg) of Co(NCS)_2_ and 2.00 mmol (194.6 µl) of 4-methyl­pyridine in 2 mL of demineralized water for 3 d at room-temperature. The violet-colored powder was filtered off and dried in air.

**Experimental details**

The X-ray powder pattern was measured using a Stoe Transmission Powder Diffraction System (STADI P) equipped with a linear, position-sensitive MYTHEN 1K detector from Stoe & Cie. Thermogravimetry and differential thermoanalysis (TG-DTA) measurements were performed in a dynamic nitro­gen atmosphere in Al_2_O_3_ crucibles with 8°C min^−1^ using a STA-PT 1000 thermobalance from Linseis. The TG-DTA instrument was calibrated using standard references materials.

## 7. Refinement

Crystal data, data collection and structure refinement details are summarized in Table 3 ▸. Hydrogen atoms were positioned with idealized geometry (methyl H atoms allowed to rotate and not to tip) and were refined with *U*_ĩso_(H) = 1.2*U*_eq_(C) (1.5 for methyl H atoms) using a riding model.

## Supplementary Material

Crystal structure: contains datablock(s) I. DOI: 10.1107/S2056989024004997/jp2006sup1.cif

Structure factors: contains datablock(s) I. DOI: 10.1107/S2056989024004997/jp2006Isup2.hkl

CCDC reference: 2358516

Additional supporting information:  crystallographic information; 3D view; checkCIF report

## Figures and Tables

**Table 1:** Selected geometric parameters (Å, °)

Co1—N2	2.091 (3)	Co1—N41	2.173 (3)
Co1—N1	2.097 (3)	Co1—N21	2.180 (3)
Co1—N11	2.162 (3)	Co1—N31	2.183 (3)
			
N2—Co1—N1	179.47 (14)	N11—Co1—N21	178.63 (12)
N2—Co1—N11	88.91 (13)	N41—Co1—N21	91.01 (12)
N1—Co1—N11	90.82 (13)	N2—Co1—N31	88.72 (13)
N2—Co1—N41	89.29 (12)	N1—Co1—N31	90.82 (12)
N1—Co1—N41	91.17 (12)	N11—Co1—N31	88.60 (12)
N11—Co1—N41	90.33 (12)	N41—Co1—N31	177.75 (12)
N2—Co1—N21	90.86 (13)	N21—Co1—N31	90.05 (12)
N1—Co1—N21	89.41 (13)		

**Table 2:** Hydrogen-bond geometry (Å, °)

*D*—H⋯*A*	*D*—H	H⋯*A*	*D*⋯*A*	*D*—H⋯*A*
C14—H14⋯S1^i^	0.95	2.89	3.692 (5)	142
C22—H22⋯S2^ii^	0.95	2.98	3.604 (4)	125
C25—H25⋯N2	0.95	2.65	3.164 (6)	114
C31—H31⋯N1	0.95	2.68	3.181 (5)	114
C35—H35⋯N2	0.95	2.65	3.129 (5)	112
C41—H41⋯N2	0.95	2.57	3.062 (5)	113

Symmetry codes: (i) 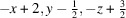 ; (ii) 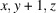 .

**Table 3:** Experimental details

Crystal data	
Chemical formula	[Co(NCS)_2_(C_6_H_7_N)_4_]
*M*_r_	547.59
Crystal system, space group	Monoclinic, *P*2_1_/*c*
Temperature (K)	200
*a*, *b*, *c* (Å)	19.0089 (7), 9.7403 (3), 16.7516 (6)
β (°)	113.370 (3)
*V* (Å^3^)	2847.15 (18)
*Z*	4
Radiation type	Mo *K*α
μ (mm^−1^)	0.77
Crystal size (mm)	0.14 × 0.10 × 0.06

Data collection	
Diffractometer	Stoe *IPDS2*
Absorption correction	Numerical (*X-RED* and *X-SHAPE*; Stoe, 2008 ▸)
*T*_min_, *T*_max_	0.735, 0.942
No. of measured, independent and observed [*I* > 2σ(*I*)] reflections	22646, 5557, 4740
*R*_int_	0.075
(sin θ/λ)_max_ (Å^−1^)	0.617

Refinement	
*R*[*F* ^2^ > 2σ(*F* ^2^)], *wR*(*F* ^2^), *S*	0.058, 0.164, 1.10
No. of reflections	5557
No. of parameters	321
H-atom treatment	H-atom parameters constrained
Δρ_max_, Δρ_min_ (e Å^−3^)	0.37, −0.34

Computer programs: *X-AREA* (Stoe, 2008 ▸), *SHELXT2014/4* (Sheldrick, 2015*a* ▸), *SHELXL2016/6* (Sheldrick, 2015*b* ▸), *DIAMOND* (Brandenburg & Putz, 1999 ▸), *XP* in *SHELXTL*-PC (Sheldrick, 2008 ▸) and *publCIF* (Westrip, 2010 ▸).

## supplementary crystallographic information

### Crystal data

**Table:**

[Co(NCS)_2_(C_6_H_7_N)_4_]	*F*(000) = 1140
*M**_r_* = 547.59	*D*_x_ = 1.277 Mg m^−^^3^
Monoclinic, *P*2_1_/*c*	Mo *K*α radiation, λ = 0.71073 Å
*a* = 19.0089 (7) Å	Cell parameters from 22661 reflections
*b* = 9.7403 (3) Å	θ = 2.4–26.0°
*c* = 16.7516 (6) Å	µ = 0.77 mm^−^^1^
β = 113.370 (3)°	*T* = 200 K
*V* = 2847.15 (18) Å^3^	Plate, red
*Z* = 4	0.14 × 0.10 × 0.06 mm

### Data collection

**Table:**

Stoe IPDS-2 diffractometer	4740 reflections with *I* > 2σ(*I*)
ω scans	*R*_int_ = 0.075
Absorption correction: numerical (X-Red and X-Shape; Stoe, 2008)	θ_max_ = 26.0°, θ_min_ = 2.4°
*T*_min_ = 0.735, *T*_max_ = 0.942	*h* = −23→23
22646 measured reflections	*k* = −12→11
5557 independent reflections	*l* = −19→20

### Refinement

**Table:**

Refinement on *F*^2^	Hydrogen site location: inferred from neighbouring sites
Least-squares matrix: full	H-atom parameters constrained
*R*[*F*^2^ > 2σ(*F*^2^)] = 0.058	*w* = 1/[σ^2^(*F*_o_^2^) + (0.0526*P*)^2^ + 3.0556*P*] where *P* = (*F*_o_^2^ + 2*F*_c_^2^)/3
*wR*(*F*^2^) = 0.164	(Δ/σ)_max_ = 0.001
*S* = 1.10	Δρ_max_ = 0.37 e Å^−^^3^
5557 reflections	Δρ_min_ = −0.34 e Å^−^^3^
321 parameters	Extinction correction: *SHELXL2016/6* (Sheldrick, 2015b), Fc^*^=kFc[1+0.001xFc^2^λ^3^/sin(2θ)]^-1/4^
0 restraints	Extinction coefficient: 0.020 (2)

### Special details

**Table:**

Geometry. All esds (except the esd in the dihedral angle between two l.s. planes) are estimated using the full covariance matrix. The cell esds are taken into account individually in the estimation of esds in distances, angles and torsion angles; correlations between esds in cell parameters are only used when they are defined by crystal symmetry. An approximate (isotropic) treatment of cell esds is used for estimating esds involving l.s. planes.

### Fractional atomic coordinates and isotropic or equivalent isotropic displacement parameters (Å^2^)

**Table:**

	*x*	*y*	*z*	*U*_iso_*/*U*_eq_	
Co1	0.75009 (3)	0.27830 (5)	0.72802 (3)	0.0598 (2)	
N1	0.83018 (19)	0.4369 (4)	0.7493 (2)	0.0687 (8)	
C1	0.8514 (2)	0.5485 (4)	0.7635 (3)	0.0700 (10)	
S1	0.88072 (10)	0.70552 (14)	0.78286 (16)	0.1355 (8)	
N2	0.6711 (2)	0.1190 (3)	0.7075 (2)	0.0706 (9)	
C2	0.6392 (2)	0.0154 (4)	0.6866 (2)	0.0627 (9)	
S2	0.59471 (9)	−0.12915 (13)	0.65766 (9)	0.0953 (4)	
N11	0.81009 (18)	0.1530 (3)	0.6687 (2)	0.0631 (8)	
C11	0.7743 (2)	0.0719 (4)	0.5998 (3)	0.0734 (11)	
H11	0.719864	0.073772	0.573384	0.088*	
C12	0.8125 (3)	−0.0137 (5)	0.5657 (3)	0.0798 (12)	
H12	0.784522	−0.067672	0.515937	0.096*	
C13	0.8911 (3)	−0.0219 (4)	0.6033 (3)	0.0773 (11)	
C14	0.9283 (2)	0.0599 (5)	0.6740 (3)	0.0729 (11)	
H14	0.982624	0.057449	0.702150	0.087*	
C15	0.8869 (2)	0.1452 (4)	0.7040 (2)	0.0674 (10)	
H15	0.914097	0.202117	0.752488	0.081*	
C16	0.9359 (4)	−0.1151 (6)	0.5673 (4)	0.114 (2)	
H16A	0.943638	−0.204637	0.596216	0.171*	
H16B	0.985779	−0.073434	0.578040	0.171*	
H16C	0.907147	−0.127082	0.504637	0.171*	
N21	0.69095 (18)	0.4019 (3)	0.7907 (2)	0.0618 (7)	
C21	0.6878 (2)	0.5394 (4)	0.7865 (3)	0.0666 (10)	
H21	0.711052	0.584993	0.753049	0.080*	
C22	0.6527 (2)	0.6173 (4)	0.8283 (3)	0.0705 (10)	
H22	0.652627	0.714535	0.823941	0.085*	
C23	0.6172 (2)	0.5543 (4)	0.8771 (3)	0.0678 (10)	
C24	0.6202 (3)	0.4139 (4)	0.8810 (3)	0.0708 (10)	
H24	0.596838	0.365610	0.913386	0.085*	
C25	0.6572 (2)	0.3426 (4)	0.8376 (3)	0.0681 (10)	
H25	0.658601	0.245226	0.841550	0.082*	
C26	0.5760 (3)	0.6355 (5)	0.9219 (3)	0.0942 (15)	
H26A	0.579480	0.733587	0.911053	0.141*	
H26B	0.599684	0.617846	0.984604	0.141*	
H26C	0.522031	0.607787	0.899158	0.141*	
N31	0.82027 (19)	0.1859 (3)	0.8531 (2)	0.0627 (8)	
C31	0.8626 (3)	0.2631 (4)	0.9211 (3)	0.0714 (11)	
H31	0.861677	0.359849	0.913605	0.086*	
C32	0.9076 (3)	0.2104 (4)	1.0015 (3)	0.0767 (12)	
H32	0.937495	0.270155	1.047220	0.092*	
C33	0.9093 (3)	0.0714 (4)	1.0156 (3)	0.0715 (10)	
C34	0.8663 (3)	−0.0092 (4)	0.9449 (3)	0.0780 (12)	
H34	0.866465	−0.106195	0.950902	0.094*	
C35	0.8234 (2)	0.0505 (4)	0.8662 (3)	0.0709 (11)	
H35	0.794590	−0.007362	0.818849	0.085*	
C36	0.9558 (3)	0.0107 (5)	1.1038 (3)	0.0936 (15)	
H36A	0.979077	0.084740	1.145571	0.140*	
H36B	0.996251	−0.047877	1.099818	0.140*	
H36C	0.922411	−0.044334	1.123206	0.140*	
N41	0.67845 (18)	0.3624 (3)	0.6016 (2)	0.0622 (8)	
C41	0.6027 (2)	0.3398 (4)	0.5677 (2)	0.0625 (9)	
H41	0.580692	0.296345	0.603153	0.075*	
C42	0.5553 (2)	0.3761 (4)	0.4845 (3)	0.0641 (9)	
H42	0.501995	0.356864	0.463767	0.077*	
C43	0.5844 (2)	0.4403 (4)	0.4309 (2)	0.0675 (10)	
C44	0.6623 (3)	0.4658 (4)	0.4658 (3)	0.0716 (10)	
H44	0.685217	0.510643	0.431756	0.086*	
C45	0.7068 (2)	0.4263 (4)	0.5496 (3)	0.0676 (10)	
H45	0.760195	0.445089	0.571864	0.081*	
C46	0.5336 (3)	0.4760 (5)	0.3387 (3)	0.0888 (14)	
H46A	0.561211	0.537278	0.314717	0.133*	
H46B	0.487385	0.522127	0.337480	0.133*	
H46C	0.519184	0.391905	0.303875	0.133*	

### Atomic displacement parameters (Å^2^)

**Table:**

	*U*^11^	*U*^22^	*U*^33^	*U*^12^	*U*^13^	*U*^23^
Co1	0.0631 (3)	0.0557 (3)	0.0541 (3)	−0.0041 (2)	0.0163 (2)	0.0001 (2)
N1	0.0677 (19)	0.064 (2)	0.067 (2)	−0.0042 (16)	0.0191 (16)	0.0020 (16)
C1	0.062 (2)	0.063 (2)	0.079 (3)	−0.0027 (19)	0.021 (2)	−0.004 (2)
S1	0.0977 (11)	0.0653 (8)	0.239 (2)	−0.0171 (7)	0.0625 (13)	−0.0333 (10)
N2	0.075 (2)	0.0621 (19)	0.068 (2)	−0.0067 (17)	0.0203 (17)	0.0009 (16)
C2	0.066 (2)	0.062 (2)	0.053 (2)	−0.0015 (18)	0.0160 (17)	0.0060 (17)
S2	0.1259 (11)	0.0697 (7)	0.0837 (8)	−0.0312 (7)	0.0345 (8)	−0.0084 (6)
N11	0.0613 (18)	0.0658 (19)	0.0539 (17)	−0.0028 (15)	0.0141 (14)	−0.0015 (14)
C11	0.068 (2)	0.077 (3)	0.065 (2)	−0.004 (2)	0.0153 (19)	−0.014 (2)
C12	0.089 (3)	0.067 (3)	0.073 (3)	−0.003 (2)	0.022 (2)	−0.015 (2)
C13	0.091 (3)	0.059 (2)	0.080 (3)	0.012 (2)	0.033 (2)	0.006 (2)
C14	0.068 (2)	0.078 (3)	0.066 (2)	0.007 (2)	0.020 (2)	0.012 (2)
C15	0.067 (2)	0.073 (2)	0.053 (2)	−0.0020 (19)	0.0145 (18)	−0.0006 (18)
C16	0.131 (5)	0.093 (4)	0.125 (5)	0.032 (3)	0.058 (4)	−0.008 (3)
N21	0.0658 (18)	0.0588 (17)	0.0574 (17)	−0.0020 (14)	0.0207 (15)	0.0045 (14)
C21	0.073 (2)	0.057 (2)	0.068 (2)	0.0022 (18)	0.026 (2)	0.0085 (18)
C22	0.080 (3)	0.058 (2)	0.070 (2)	0.0021 (19)	0.026 (2)	0.0033 (18)
C23	0.075 (2)	0.066 (2)	0.059 (2)	0.0032 (19)	0.0232 (19)	0.0008 (18)
C24	0.082 (3)	0.070 (2)	0.062 (2)	0.001 (2)	0.030 (2)	0.0066 (19)
C25	0.079 (3)	0.058 (2)	0.065 (2)	−0.0021 (19)	0.026 (2)	0.0065 (18)
C26	0.121 (4)	0.079 (3)	0.097 (4)	0.003 (3)	0.057 (3)	−0.009 (3)
N31	0.0714 (19)	0.0558 (17)	0.0531 (17)	−0.0022 (15)	0.0164 (15)	−0.0030 (14)
C31	0.096 (3)	0.056 (2)	0.052 (2)	0.001 (2)	0.019 (2)	−0.0023 (17)
C32	0.094 (3)	0.071 (3)	0.053 (2)	0.002 (2)	0.016 (2)	−0.0047 (19)
C33	0.080 (3)	0.070 (2)	0.057 (2)	0.008 (2)	0.0187 (19)	0.0062 (19)
C34	0.085 (3)	0.060 (2)	0.073 (3)	0.002 (2)	0.013 (2)	0.010 (2)
C35	0.078 (3)	0.055 (2)	0.066 (2)	0.0002 (19)	0.013 (2)	0.0036 (18)
C36	0.106 (4)	0.091 (3)	0.061 (3)	0.007 (3)	0.010 (2)	0.017 (2)
N41	0.0608 (17)	0.0644 (18)	0.0550 (17)	−0.0005 (14)	0.0161 (14)	0.0022 (14)
C41	0.062 (2)	0.062 (2)	0.059 (2)	−0.0001 (17)	0.0191 (17)	0.0001 (17)
C42	0.060 (2)	0.061 (2)	0.062 (2)	0.0016 (17)	0.0150 (17)	−0.0054 (17)
C43	0.074 (2)	0.063 (2)	0.056 (2)	0.0111 (19)	0.0151 (19)	−0.0039 (17)
C44	0.080 (3)	0.069 (2)	0.064 (2)	0.000 (2)	0.026 (2)	0.0048 (19)
C45	0.064 (2)	0.070 (2)	0.064 (2)	−0.0034 (19)	0.0199 (19)	0.0067 (19)
C46	0.092 (3)	0.100 (3)	0.057 (2)	0.019 (3)	0.011 (2)	0.005 (2)

### Geometric parameters (Å, º)

**Table:**

Co1—N2	2.091 (3)	C24—H24	0.9500
Co1—N1	2.097 (3)	C25—H25	0.9500
Co1—N11	2.162 (3)	C26—H26A	0.9800
Co1—N41	2.173 (3)	C26—H26B	0.9800
Co1—N21	2.180 (3)	C26—H26C	0.9800
Co1—N31	2.183 (3)	N31—C35	1.334 (5)
N1—C1	1.151 (5)	N31—C31	1.336 (5)
C1—S1	1.616 (4)	C31—C32	1.376 (6)
N2—C2	1.158 (5)	C31—H31	0.9500
C2—S2	1.614 (4)	C32—C33	1.372 (6)
N11—C11	1.341 (5)	C32—H32	0.9500
N11—C15	1.342 (5)	C33—C34	1.385 (6)
C11—C12	1.370 (6)	C33—C36	1.509 (6)
C11—H11	0.9500	C34—C35	1.375 (5)
C12—C13	1.375 (6)	C34—H34	0.9500
C12—H12	0.9500	C35—H35	0.9500
C13—C14	1.368 (6)	C36—H36A	0.9800
C13—C16	1.523 (7)	C36—H36B	0.9800
C14—C15	1.370 (6)	C36—H36C	0.9800
C14—H14	0.9500	N41—C41	1.340 (5)
C15—H15	0.9500	N41—C45	1.345 (5)
C16—H16A	0.9800	C41—C42	1.373 (5)
C16—H16B	0.9800	C41—H41	0.9500
C16—H16C	0.9800	C42—C43	1.378 (6)
N21—C25	1.327 (5)	C42—H42	0.9500
N21—C21	1.341 (5)	C43—C44	1.382 (6)
C21—C22	1.372 (6)	C43—C46	1.501 (5)
C21—H21	0.9500	C44—C45	1.376 (5)
C22—C23	1.392 (6)	C44—H44	0.9500
C22—H22	0.9500	C45—H45	0.9500
C23—C24	1.369 (6)	C46—H46A	0.9800
C23—C26	1.506 (6)	C46—H46B	0.9800
C24—C25	1.384 (6)	C46—H46C	0.9800
			
N2—Co1—N1	179.47 (14)	N21—C25—C24	124.0 (4)
N2—Co1—N11	88.91 (13)	N21—C25—H25	118.0
N1—Co1—N11	90.82 (13)	C24—C25—H25	118.0
N2—Co1—N41	89.29 (12)	C23—C26—H26A	109.5
N1—Co1—N41	91.17 (12)	C23—C26—H26B	109.5
N11—Co1—N41	90.33 (12)	H26A—C26—H26B	109.5
N2—Co1—N21	90.86 (13)	C23—C26—H26C	109.5
N1—Co1—N21	89.41 (13)	H26A—C26—H26C	109.5
N11—Co1—N21	178.63 (12)	H26B—C26—H26C	109.5
N41—Co1—N21	91.01 (12)	C35—N31—C31	116.4 (3)
N2—Co1—N31	88.72 (13)	C35—N31—Co1	122.4 (3)
N1—Co1—N31	90.82 (12)	C31—N31—Co1	121.2 (3)
N11—Co1—N31	88.60 (12)	N31—C31—C32	123.7 (4)
N41—Co1—N31	177.75 (12)	N31—C31—H31	118.2
N21—Co1—N31	90.05 (12)	C32—C31—H31	118.2
C1—N1—Co1	154.4 (3)	C33—C32—C31	120.0 (4)
N1—C1—S1	179.7 (5)	C33—C32—H32	120.0
C2—N2—Co1	162.3 (4)	C31—C32—H32	120.0
N2—C2—S2	180.0 (5)	C32—C33—C34	116.5 (4)
C11—N11—C15	115.9 (4)	C32—C33—C36	121.3 (4)
C11—N11—Co1	123.2 (3)	C34—C33—C36	122.2 (4)
C15—N11—Co1	120.7 (3)	C35—C34—C33	120.3 (4)
N11—C11—C12	123.0 (4)	C35—C34—H34	119.8
N11—C11—H11	118.5	C33—C34—H34	119.8
C12—C11—H11	118.5	N31—C35—C34	123.1 (4)
C11—C12—C13	120.3 (4)	N31—C35—H35	118.4
C11—C12—H12	119.9	C34—C35—H35	118.4
C13—C12—H12	119.9	C33—C36—H36A	109.5
C14—C13—C12	117.2 (4)	C33—C36—H36B	109.5
C14—C13—C16	120.7 (5)	H36A—C36—H36B	109.5
C12—C13—C16	122.0 (5)	C33—C36—H36C	109.5
C13—C14—C15	119.7 (4)	H36A—C36—H36C	109.5
C13—C14—H14	120.2	H36B—C36—H36C	109.5
C15—C14—H14	120.2	C41—N41—C45	116.2 (3)
N11—C15—C14	123.8 (4)	C41—N41—Co1	120.2 (3)
N11—C15—H15	118.1	C45—N41—Co1	123.3 (3)
C14—C15—H15	118.1	N41—C41—C42	123.4 (4)
C13—C16—H16A	109.5	N41—C41—H41	118.3
C13—C16—H16B	109.5	C42—C41—H41	118.3
H16A—C16—H16B	109.5	C41—C42—C43	120.5 (4)
C13—C16—H16C	109.5	C41—C42—H42	119.8
H16A—C16—H16C	109.5	C43—C42—H42	119.8
H16B—C16—H16C	109.5	C42—C43—C44	116.5 (4)
C25—N21—C21	116.4 (4)	C42—C43—C46	121.0 (4)
C25—N21—Co1	120.5 (3)	C44—C43—C46	122.5 (4)
C21—N21—Co1	123.2 (3)	C45—C44—C43	120.2 (4)
N21—C21—C22	123.1 (4)	C45—C44—H44	119.9
N21—C21—H21	118.5	C43—C44—H44	119.9
C22—C21—H21	118.5	N41—C45—C44	123.3 (4)
C21—C22—C23	120.2 (4)	N41—C45—H45	118.4
C21—C22—H22	119.9	C44—C45—H45	118.4
C23—C22—H22	119.9	C43—C46—H46A	109.5
C24—C23—C22	116.6 (4)	C43—C46—H46B	109.5
C24—C23—C26	121.4 (4)	H46A—C46—H46B	109.5
C22—C23—C26	122.0 (4)	C43—C46—H46C	109.5
C23—C24—C25	119.8 (4)	H46A—C46—H46C	109.5
C23—C24—H24	120.1	H46B—C46—H46C	109.5
C25—C24—H24	120.1		
			
C15—N11—C11—C12	−0.6 (6)	C35—N31—C31—C32	−0.3 (7)
Co1—N11—C11—C12	−175.9 (3)	Co1—N31—C31—C32	179.7 (4)
N11—C11—C12—C13	1.5 (7)	N31—C31—C32—C33	−1.4 (8)
C11—C12—C13—C14	−1.0 (7)	C31—C32—C33—C34	2.3 (7)
C11—C12—C13—C16	−179.7 (5)	C31—C32—C33—C36	−177.9 (5)
C12—C13—C14—C15	−0.2 (6)	C32—C33—C34—C35	−1.5 (7)
C16—C13—C14—C15	178.5 (4)	C36—C33—C34—C35	178.6 (5)
C11—N11—C15—C14	−0.7 (6)	C31—N31—C35—C34	1.1 (7)
Co1—N11—C15—C14	174.7 (3)	Co1—N31—C35—C34	−178.9 (4)
C13—C14—C15—N11	1.1 (7)	C33—C34—C35—N31	−0.2 (8)
C25—N21—C21—C22	0.5 (6)	C45—N41—C41—C42	1.2 (6)
Co1—N21—C21—C22	−177.8 (3)	Co1—N41—C41—C42	−172.4 (3)
N21—C21—C22—C23	−0.8 (6)	N41—C41—C42—C43	−0.5 (6)
C21—C22—C23—C24	0.5 (6)	C41—C42—C43—C44	−0.4 (6)
C21—C22—C23—C26	−178.2 (4)	C41—C42—C43—C46	177.8 (4)
C22—C23—C24—C25	0.0 (6)	C42—C43—C44—C45	0.6 (6)
C26—C23—C24—C25	178.7 (4)	C46—C43—C44—C45	−177.5 (4)
C21—N21—C25—C24	0.0 (6)	C41—N41—C45—C44	−0.9 (6)
Co1—N21—C25—C24	178.4 (3)	Co1—N41—C45—C44	172.4 (3)
C23—C24—C25—N21	−0.3 (7)	C43—C44—C45—N41	0.0 (7)

### Hydrogen-bond geometry (Å, º)

**Table:**

*D*—H···*A*	*D*—H	H···*A*	*D*···*A*	*D*—H···*A*
C14—H14···S1^i^	0.95	2.89	3.692 (5)	142
C22—H22···S2^ii^	0.95	2.98	3.604 (4)	125
C25—H25···N2	0.95	2.65	3.164 (6)	114
C31—H31···N1	0.95	2.68	3.181 (5)	114
C35—H35···N2	0.95	2.65	3.129 (5)	112
C41—H41···N2	0.95	2.57	3.062 (5)	113

Symmetry codes: (i) −*x*+2, *y*−1/2, −*z*+3/2; (ii) *x*, *y*+1, *z*.
